# EEG components of inhibitory control ability in internet gaming disorder: A systematic review and meta‐analysis of randomized controlled trials

**DOI:** 10.1002/brb3.3388

**Published:** 2024-01-24

**Authors:** Junjian Yu, Mohammad Farris Iman Leong Abdullah, Nor Shuhada Mansor

**Affiliations:** ^1^ Department of Community Health Advanced Medical and Dental Institute Universiti Sains Malaysia Penang Malaysia

**Keywords:** EEG components, inhibitory control ability, internet gaming disorder, meta‐analysis, systematic review

## Abstract

**Background:**

Inhibitory control ability is a crucial cognitive function that enables individuals to regulate their impulses and behaviors in a goal‐directed manner. However, with the increasing prevalence of internet gaming disorder (IGD), there has been growing concern about the impact of excessive gaming on inhibitory control ability. Despite the accumulating evidence on this topic, the research conclusion on whether people with IGD have worse inhibition control ability than healthy controls remains inconsistent, and the lack of effective electroencephalography prediction indicators further complicates this issue. To address this research gap, the present study aimed to investigate whether N2 event‐related potential (ERP) and P3 ERP components could serve as reliable indicators of inhibitory control ability in individuals with IGD.

**Methods:**

To achieve this goal, a systematic literature search was conducted in several databases, including Web of Science, ScienceDirect (EBSCO), SpringerLink, PubMed, and Wiley Online Library. The inclusion criteria were strictly implemented to ensure the quality of the studies included in the meta‐analysis. In the end, a total of 5 studies, with 139 participants diagnosed with IGD and 139 healthy controls, were included in the analysis.

**Results:**

Meta‐analysis revealed large effect sizes of N2 and P3 amplitudes in individuals with IGD, indicating that these two ERP components could be potential indicators of inhibitory control ability. Specifically, the N2 and P3 amplitude was significantly larger in individuals with IGD than in the healthy control group, suggesting deficits in inhibitory control function and increased impulsivity in the IGD group. In the inhibition control task, the IGD group required more cognitive resources to suppress impulsive responses.

**Conclusion:**

Overall, the findings of this meta‐analysis shed light on the potential use of N2 and P3 amplitudes as reliable indicators of inhibitory control ability in individuals with IGD. The results provide crucial insights into the neural mechanisms underlying inhibitory control impairment in IGD, which could inform the development of effective interventions for this condition. Further research is needed to explore the functional significance of these ERP components and their potential clinical applications in the diagnosis and treatment of IGD.

## Graphical abstract

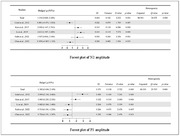

## 1 INTRODUCTION

Ever since the world entered the era of internet information, the internet has become an indispensable and vital component of our daily lives. As the internet continues to gain popularity and the gaming industry experiences rapid growth, psychological issues related to internet gaming are increasingly being acknowledged as a global concern (Brailovskaia et al., 2022; Wang & Cheng, 2022; Yao et al., 2022; Yuan et al., 2022). Internet gaming disorder (IGD) is a mental health condition characterized by excessive use of and addiction to online gaming, leading to psychological and social impairments, such as sleep disorders, anxiety, depression, decreased academic and professional performance, and even suicidal behavior (Madran & Akilci, 2014; Petry et al., 2015). Additionally, persistent preoccupation with internet gaming, withdrawal symptoms when not engaged in gaming, tolerance, that is, the need to spend more time playing to achieve the same satisfaction, and an inability to control gaming behavior despite negative consequences were among the features of IGD (Petry et al., 2014; Young, 1998). This disorder is included in the appendix of the Diagnostic and Statistical Manual of Mental Disorders (5th edition, DSM‐5). In mid‐2018, the World Health Organization (WHO) adopted the 11th revision of the International Classification of Diseases (ICD‐11) at the 72nd World Health Assembly. Gaming disorder, encompassing both online and offline variants, has been included in the ICD‐11 as a clinically recognizable and significant syndrome. This classification is applied when the pattern of gaming behavior is of such nature and intensity that it results in marked distress or significant impairment in personal, family, social, educational, or occupational functioning (World Health Organization, 2018). A systematic review of 37 cross‐sectional studies revealed that the global prevalence of IGD ranges between 0.7% and 27.5%; the prevalence rate is often higher in males, young people, and clinical population (Darvesh et al., 2020; Mihara & Higuchi, 2017). As a result, IGD has become a focal point for psychologists and sociological researchers.

Inhibitory control ability is a key cognitive function that plays an essential role in regulating behavior and preventing impulsive actions (Dong et al., 2019; Yao et al., 2022). This ability refers to an individual's capacity to suppress irrelevant information and stimuli when striving to achieve a specific goal (Rothbart & Posner, 1985). During cognitive processing, individuals must allocate their limited cognitive resources to the current target task, necessitating the prevention of irrelevant information from entering working memory or inhibiting predominant but task‐independent behaviors or reactions (Logan et al., 2010). It has been suggested that IGD may be associated with deficits in inhibitory control ability (Dong et al., 2019; Yao et al., 2022) resulting in persistent and impulsive engagement in long‐duration gaming. Currently, various experimental paradigms for studying inhibitory function have demonstrated that online gaming addicts perform worse in inhibitory control function tasks compared to control groups. For instance, a study on the classic color‐word Stroop task discovered that internet gaming subjects took longer to complete the task than the healthy control group, and they made more errors, indicating impaired inhibitory control function among the internet‐addicted subjects (Dong et al., 2011; Jeromin, Rief, & Barke, 2016; Zhou et al., 2012). The Go/NoGo experiment revealed that the group with online gaming disorder exhibited lower NoGo accuracies than the control group. This difference in performance could be attributed to behavioral inhibition failure and weakened conflict control, which require more cognitive resources to complete suppression responses under certain circumstances (Chen et al., 2022). The response inhibition abilities of individuals with gaming disorder are weakened, which might be attributed to the failure of behavioral inhibition and reduced conflict control, ultimately requiring more cognitive resources to complete response suppression under specific conditions (Chen et al., 2022; Fathi et al., 2022). A meta‐analysis found that, compared to healthy individuals, those with gaming disorders were more likely to display impaired response inhibition (Argyriou et al., 2017). The research findings suggest that impaired inhibition control often manifests as impulsivity and a lack of self‐regulation ability (Smith et al., 2014; Snyder et al., 2015). Dong and Potenza (2014) proposed that individuals with IGD may exhibit impaired inhibition control in the form of intense cravings for gaming rewards and uncontrollable internet usage.

Recent studies have focused on the neurobiological mechanisms underlying inhibitory control deficits in individuals with IGD (Dong et al., 2020). Electroencephalography (EEG), a noninvasive method to explore the neural correlates of IGD, provides a high temporal resolution to study the event‐related potentials (ERPs) associated with inhibitory control in this population (Hegerl et al., 2008; Liu et al., 2022). Two ERP components that have been widely used to investigate inhibitory control are the N2 and P3 components (Dustman et al., 1996; Wu et al., 2022). The N2 component is a negative deflection that occurs approximately 200–300 ms after stimulus onset and is thought to reflect the allocation of attention to the task‐relevant stimulus and the inhibition of irrelevant information (Folstein & Van Petten, 2008). The P3 component is a positive deflection that occurs approximately 300–500 ms after stimulus onset and is thought to reflect the allocation of attention to the task‐relevant stimulus and the updating of working memory (Polich, 2007). The amplitudes of N2 and P3 are commonly used to evaluate inhibitory control ability. A meta‐analysis and an independent EEG experiment found that correlations among the N2 latency, P3 latency, and the stop‐signal reaction time are indeed replicable. Both N2 and P3 latency measures show similar or even higher correlations with other inhibitory control behavioral parameters such as the go reaction time or stopping accuracy (Huster et al., 2020). A Go/NoGo study reported that both components N2 and P3 are enhanced when a response is withheld (no‐go trial) within a series of positive responses (go trials) and appeared abnormal monitoring performance in the anterior cingulate cortex (ACC) and adjacent brain regions (Bekker et al., 2005; Bokura et al., 2001). The ACC and adjacent regions are also involved in the generation of the frontal ERP N2 and P3 components (Veen & Carter, 2002). In cognitive neuroscience, two ERP components N2 appearing in the frontal area of the scalp EEG experiment and P3 appearing in the frontal area were associated with inhibition, which appeared successfully over frontal electrodes with increasing demand of inhibition control in NoGo or stop trials during inhibition tasks, and have been considered an index of inhibition control (Albert et al., 2013; Donkers &Van Boxtel, 2004; Folstein & Van Petten, 2008).

Previous studies have also shown that the amplitudes of N2 and P3 components are related to inhibitory control ability in individuals with IGD (Liu et al., 2022). Meanwhile, some studies have found a correlation between N2 and P3 EEG components and the inhibition control of IGD behavior through correlation analysis. Kim et al. (2017) found that significant relationships were found between NoGo‐N2 latency at Cz and internet addition test (IAT) scores (*r* = .452, *p* = .018) in the IGD group. NoGo‐N2 latency at C2 correlated with IAT scores (*r* = .057, *p* = .777) in the IGD group. Li et al. (2019) revealed that the correlation between NoGo N2 and inhibitory control in internet gaming addiction was −0.24, and the correlation between NoGo P3 and inhibitory control in internet gaming addiction was −0.20. Moreover, studies in the Go/NoGo experiments have identified a correlation between deficient inhibitory control in individuals with internet addiction disorder and reduced NoGo N2 amplitude as well as increased NoGo P3 amplitude (Dong et al., 2010; Zhou et al., 2010). Kim et al. (2017) observed delayed NoGo‐N2 latency in individuals with IGD when compared to healthy control subjects, indicating that individuals with IGD encounter difficulties in early‐stage response inhibition, which necessitates higher cognitive resources. The severities of IGD and impulsivity were positively associated with NoGo‐N2 latency at the central site, suggesting that the impaired inhibitory control in individuals with IGD may be attributed to an elevated cognitive demand for response inhibition due to their heightened impulsivity. Fathi et al. (2022), through stop‐signal experiments, noted that in stop trials, the video game addiction (VGA) group exhibited reduced N2 amplitude compared to the control group in proactive stop trials. Additionally, the VGA group demonstrated an increased P3 amplitude in the proactive stop trials. The variations in P3 amplitude during the stop trials may be attributed to reflect the attentional resources required for subjects to respond accurately to anticipated targets, which could potentially signify compensatory mechanisms in response or deficiencies in impulse control among VGA subjects.

However, the results of previous studies on the association between inhibitory control and N2/P3 components in individuals with IGD have been inconsistent. These studies have produced mixed results, with some showing that the N2 and P3 amplitudes are higher in individuals with IGD, whereas others have reported no significant differences in N2/P3 amplitudes compared to healthy controls. On the one hand, some studies suggest individuals with IGD may have an increased need for cognitive resources to inhibit inappropriate responses, demonstrated by larger N2 and P3 amplitudes during the Go/NoGo task (Badzakova‐Trajkov et al., 2009; Kiefer et al., 1998). On the other hand, studies did not find any differences in N2/P3 amplitudes between individuals with IGD and healthy controls. For example, Littel and Franken (2012) found that the amplitudes of the frontal–central N2 and central–parietal P3 components in response to NoGo stimuli did not differ between internet gamers and controls. The contradicting literature on neurophysiological markers, therefore, warranted a systematic review and meta‐analysis to investigate the relations between N2/P3 amplitudes and inhibitory control ability in IGD. Thus, the present review seeks to determine whether N2/P3 ERP components could serve as reliable indicators of inhibitory control ability in individuals with IGD.

## 2 METHODS

### 2.1 Search strategy

A comprehensive search in Web of Science, ScienceDirect, PubMed, Springer, and ProQuest was independently performed by authors through March 1st, 2023, using the following search terms: (“inhibitory control”) AND (“Internet game addiction” OR Internet game disorder OR “Online game addiction” OR “Video game addiction”) AND (“Event‐Related Potentials” OR “ERP”). The study authors compared their list of studies for the review and reached a consensus regarding selected studies to be included in the analysis.

### 2.2 Inclusion and exclusion criteria

To be eligible for review inclusion, studies must meet the following criteria as follows:

1. Participants were individuals with IGD. IGD individuals are characterized by playing games for more than 4 h a day in their daily lives, playing games for more than a year, and being identified as having symptoms of internet gaming addiction or IGD. Their condition was evaluated using validated questionnaires (e.g., DSM‐5, Internet Addiction Test, Internet Game Disorder Questionnaire, and Young's Diagnostic Questionnaire for Internet Addiction). The domains evaluated by these questionnaires were primarily excessive use and addiction to online games.
2. Healthy controls were used comparative group.
3. Used standard inhibitory control tasks, such as the Stroop task, the Go/NoGo task, the stop signal task, the flanker task, and the Simon task.
4. Record brain activity using scalp EEG reporting on N2 and P3 amplitude.
5. Written in the English language and published in a peer‐reviewed journal.

**FIGURE 1:**
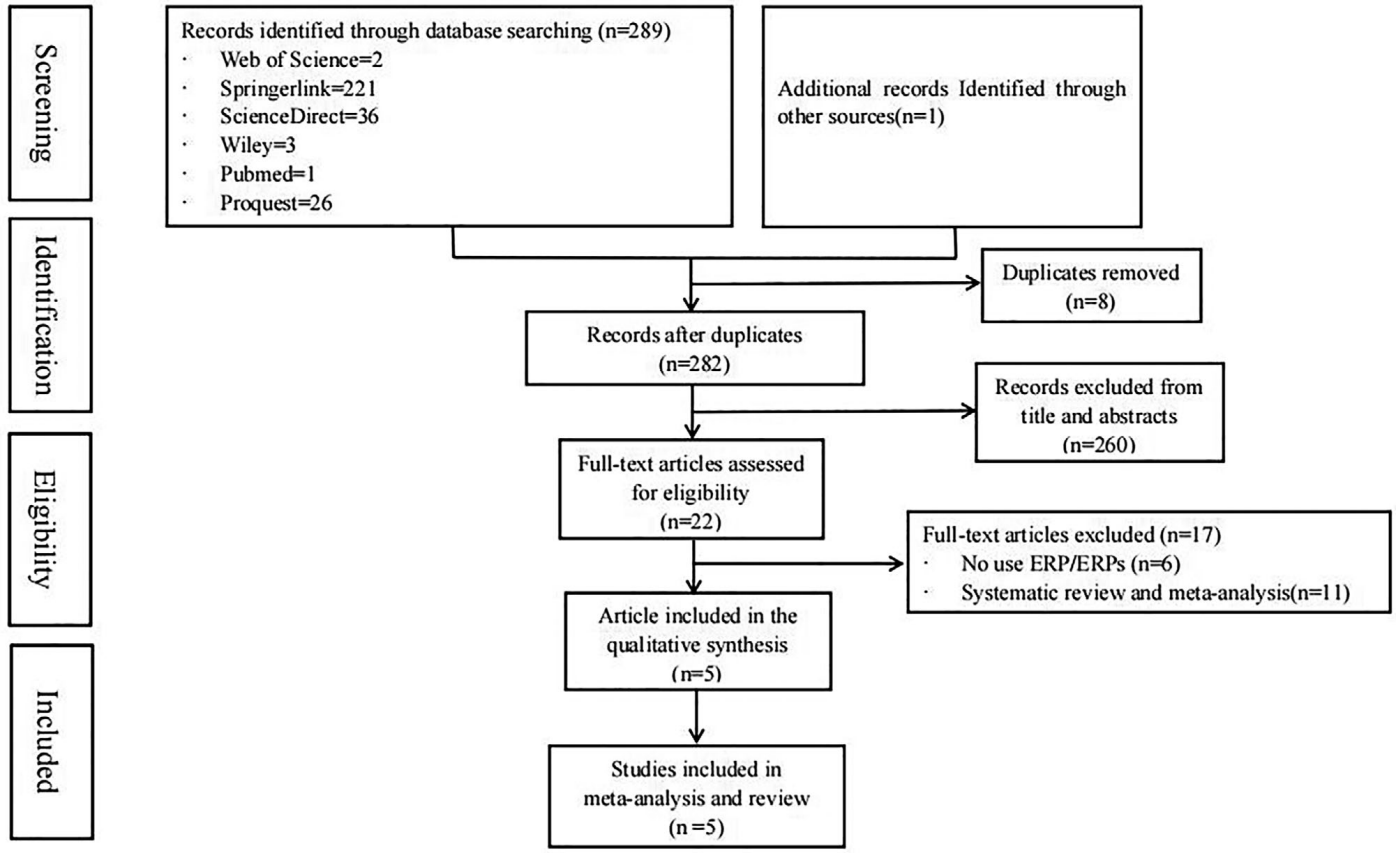
PRISMA flow chart diagram showing the process of study selection for inclusion in the systematic review and meta‐analysis.

However, non‐EEG studies (i.e., fNIRS, MRI, and fMRI) were not eligible for review. Additionally, studies using participants with a history of mental illness, mental disorder, brain injury or who have taken psychiatric drugs in the past and have blindness or color weakness, visual impairment, or hearing impairment would be excluded. Figure 1 illustrates the process of study selection for inclusion in the review.

### 2.3 Data extraction

Data extracted included sample characteristics (sample size, age, and gender), internet gaming duration, assessment of IGD (questionnaires) as well as the internet gaming behavior indicators related to inhibitory control (experimental paradigm of inhibitory control and amplitudes of N2 and P3 components) for a comprehensive statistical assessment of inhibitory control with IGD individuals. Additionally, EEG information, such as EEG equipment, EEG channels, EEG reference, stimulus duration (ms), and electrodes, are also included. Amplitude refers to the magnitude or strength of the electrical signal recorded at specific electrode sites, whereas latency refers to the time it takes for the component to reach its peak or specific point of interest. According to Luck and Gaspelin (2017), amplitude measures provide information about the magnitude of neural activation associated with a specific cognitive process. A larger amplitude typically indicates a stronger or more pronounced brain response researchers can assess differences in cognitive processing by comparing amplitudes between conditions or groups. Furthermore, amplitude measures of N2 and P3 components tend to exhibit good test–retest reliability, as noted by Cuthbert et al. (2000), indicating that they can be consistently measured across different experimental sessions or subjects. This reliability makes amplitude a robust measure for detecting changes in neural activity. Although frequency analysis can also provide valuable insights into patterns of neural synchronization and communication between brain regions, it typically complements the analysis of ERPs. Amplitude analysis, on the other hand, is commonly used and can provide information regarding the intensity and differences in cognitive processes (Luck, 2014; Luck & Gaspelin, 2017). In summary, amplitude analysis is favored when studying N2 and P3 components due to its ability to provide information about neural activation magnitude, its reliability in measuring changes in neural activity, and its common usage in cognitive neuroscience research. Frequency analysis, although valuable in revealing neural synchronization patterns, is often complementary to the analysis of ERPs.

### 2.4 Quality assessment

The Cochrane Collaboration risk‐of‐bias tool 2.0 was used to evaluate the methodological quality and risk of bias of the five studies analyzed. The included studies’ quality was evaluated based on the following aspects: (a) Randomization process; (b) timing of identification or recruitment of participants; (c) deviations from the intended interventions; (d) missing outcome data; (e) measurement of the outcome, selection of the reported result; (f) overall bias.

### 2.5 Publication bias

Publication bias refers to the phenomenon that in similar studies, papers with positive results (studies with statistically significant results) are more likely to be accepted and published than papers with negative results (studies with no statistically significant results) (Rothstein et al., 2005). This study used the fail‐safe number (*Nfs*), funnel plot, and Begg and Mazumdar rank correlation test to examine the publication bias in the quality included in the meta‐analysis. The *Nfs* is used to estimate how many unpublished quality need to be included, which can change the research results from significant to insignificant, in order to test for publication bias according to Rosenthal's suggestion, the loss of safety factor *(Nfs)* is greater than 5*k*+10 (*k* is the number of studies), indicating that meta‐analysis publication has been effectively controlled (Orwin, 1993; Rothstein et al., 2005); for funnel plots, if the graph presents a symmetrical inverted funnel shape, it indicates that the publication deviation is small and has a small impact on the meta‐analysis results (Light & Pillemer, 1984); The Begg and Mazumdar rank correlation is not significant, indicating that the meta‐analysis results are relatively stable and the possibility of serious publication bias is low.

### 2.6 Statistical analysis

The meta‐analysis strategy used comprehensive meta‐analysis CMA 3.0 (CMA; Borenstein et al., 2013; Higgins & Green, 2005; Higgins et al., 2019). In the primary analysis, the overall effect size was represented by Hedges’ *g* to make the report clearer. The Hedges’ *g* effect size and variance are based on the number of participants and the *F* value of the two groups. The Hedges’ *g* effect size is categorized as small (effect size = 0.2), medium (effect size = 0.50), or large (effect size = 0.80). The current review used a random effect model to estimate between group differences as varying sample characteristics and random errors may affect EEG observation in included studies. These guidelines are employed to assess the effect size of relationships reported in the meta‐analysis. Then, the *Q* statistics (statistical testing of heterogeneity) and the *I* ^2^ index (representing the degree of heterogeneity) were used to evaluate the extent of heterogeneity of respective effect sizes (Higgins et al., 2019). The range of *I* ^2^ values was 0%–100%, of which 25% represents low heterogeneity, 50% represents medium intensity heterogeneity, and 75% represents high heterogeneity (Higgins et al., 2003). In meta‐analysis, “heterogeneity” refers to the differences among the study results. “Low heterogeneity” indicates that the study results have minimal differences, and the findings from each study tend to be consistent. “Medium intensity heterogeneity” suggests that there is some degree of difference among the study results, but these differences are not significant, and there is still a certain level of consistency among the results. “Highly heterogeneous” refers to the situation where the studies included in the analysis exhibit variability or diversity in their findings or effect sizes (Higgins et al., 2003). High heterogeneity can occur between different studies and may be attributed to various factors such as differences in effect sizes, study designs, populations, sample characteristics, or other factors included in the studies (Imrey, 2020). Therefore, it is important to further investigate the sources of heterogeneity to analyze their impact on the research outcomes.

## 3 RESULTS

### 3.1 Study characteristics

After excluding studies according to our predefined criteria, five articles totaling 139 IGD participants and 139 healthy control group participants were included in the analyses. The average age of IGD participants ranged from 15.81 to 26.5 years old, whereas the average age of control group participants ranged from 15.91 to 24.7 years old. The average age of participants in the IGD and control groups was similar. The participants in the IGD group are mostly male, with a proportion ranging from 54% to 100%, whereas the proportion of male participants in the control group ranges from 37% to 100%. The IGD group consisted of approximately 25–31 participants, with an average online time of over 4 h per day. The subjects included in the control group were concentrated around 24–32 people, and the average daily online time of the subjects was mostly concentrated at half an hour. IGD patients often use the Diagnostic and Statistical Manual of Mental Disorders, fifth edition (DSM‐5), IAT, video game addition test, and the internet game disorder questionnaire (IGD) for evaluation. The experimental paradigm of inhibition control mainly adopts the Go/NoGo experiment, and the task stimulation time in the experiment ranges from 300 to 1000 ms. In addition, by comparing the selection of EEG devices, EEG references, EEG selection references, and electrode points in different experiments, we found that the selection of EEG devices and EEG channels is diverse EEG devices mainly involve BioSemis produced in the Netherlands, Neuroscan produced in the United States, Mitsur produced in Russia, and Brain Products produced in Germany. EEG channels range from 32 to 128, and EEG references are generally used linked mastoid for reference, and the selection of EEG electrode points is mostly concentrated in the front, central, and parallel brain regions. The primary studies were conducted until March 30, 2023. All studies have experimental designs studies (Table 1).

**TABLE 1:** Characteristics of the reviewed studies.

	IGD				Control						EEG				
	*N*	Age (Mean ± SD)	GT (hours/day)	Male (%)	*N*	Age (Mean ± SD)	GT (hours/day)	Male (%)	Questionnaire	Task paradigm	Equipment	Channels	Reference	Stimulus duration (ms)	Electrodes
Littel et al. (2012)	25	20.52 ± 2.95	4.67 ± 2.29	92	27	21.42 ± 2.59	0.47 ± 1.20	37	CIUS	Go/NoGo	BioSemi, The Netherlands	32	Linked mastoid	700	Fz, FCz, Cz, CPz, and Pz
Kim et al. (2017)	27	26.5 ± 6.1	4	89	26	24.7 ± 4.7	NA	69	DSM‐5 IAT	Go/NoGo	Neuroscan, USA	128	Linked mastoid	300	F1, Fz, F2, C1, Cz, C2 for N2
															C1, Cz, C2, P1, Pz, P2 for P3
Li et al. (2020)	31	15.81 ± 1.68	4.52 ± 3.54	90	32	15.91 ± 1.73	0.36 ± 0.50	90	YDQ	Go/NoGo	Neuroscan, USA	64	Linked mastoid	500	Fz, FCz, Cz, CPz, Pz
Fathi et al. (2022)	30	20.39 ± 3.03	5.72 ± 1.24	100	30	19.91 ± 1.94	1.34 ± 1.11	100	VAT	Stop signal task	Mitsar, Russia	32	NA	500	Fz, F3, F4, Cz, C3, C4
Chen et al. (2022)	26	20.15 ± 1.69	>2 h per day for >1 year	54	24	20.17 ± 1.44	<0.29 h per day	50	IAT IGD	Go/NoGo	Brain Product, Germany	64	Linked mastoid	1000	F3, F4, Fz, FC3, FC4, CZ, C1, C2, C3, C4, CP1, CP2, CP3, CP4

Abbreviations: CIUS, compulsive internet use scale; DSM‐5, Diagnostic and Statistical Manual of Mental Disorders, fifth edition; EEG, electroencephalography; IAT, internet addiction test; IGD, internet game disorder questionnaire; VAT, video game addiction test; YDQ, Young's diagnostic questionnaire for internet addiction.

### 3.2 Quality assessment

**FIGURE 2:**
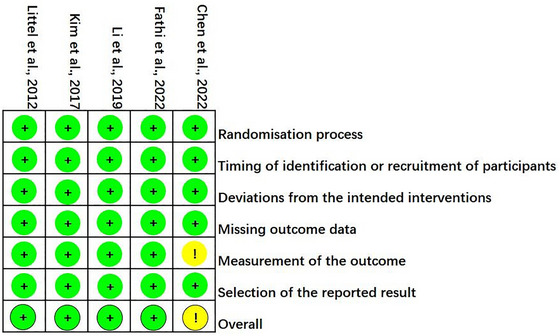
Risk of bias summary.

**FIGURE 3:**
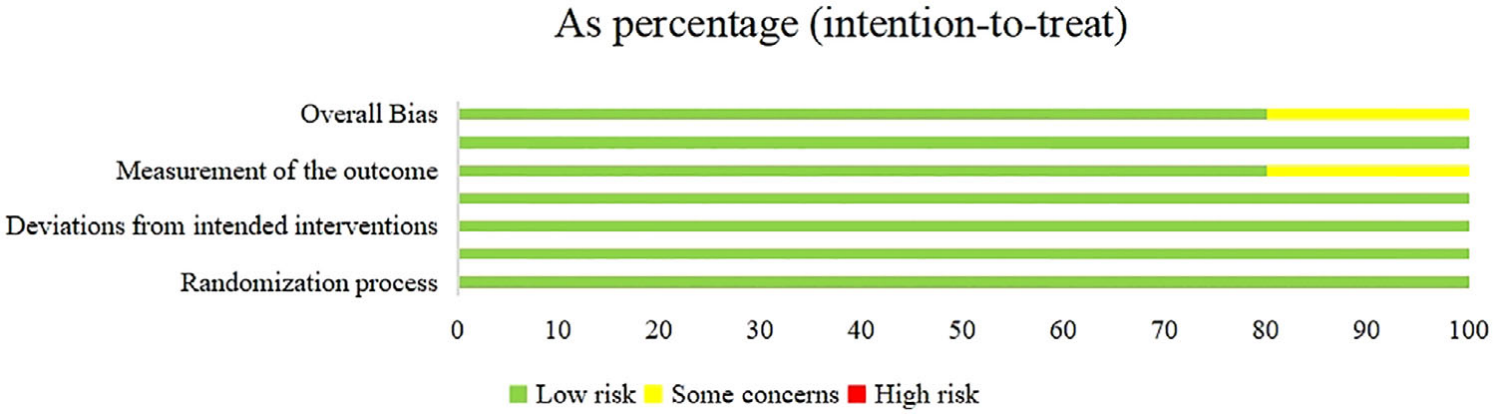
Risk of bias graph.

Our findings regarding methodological quality and risk of bias are presented in the risk of bias summary (Figure 2) and risk of bias graph (Figure 3). In the five included studies, the reporting risks of bias and other biases were assessed as low.

### 3.3 Event‐related potential for inhibitory tasks

Meta‐analysis suggests a large mean difference in N2 amplitude (Hedges’ *g* = 1.510, *p* = .001, 95% CI = .600–2.420) between IGD and healthy controls. This result indicates that the N2 amplitude of the IGD group is significantly higher than the control group during inhibitory control tasks. However, the mean differences are highly heterogeneous (*I* ^2^ = 88.911%, *Q* = 36.072, *p* < .001) suggesting a high between‐studies variation. Furthermore, we conducted a sensitivity analysis. In the analysis of the N2 EEG component, it was observed that the effect size from Li et al. (2019) deviated the most from other effect sizes, with Hedges’ *g* value of 2.613 and an effect size range of 1.937–3.289. Upon removing the data from this study, we found that the remaining four studies had an effect size with Hedges’ *g* value of 1.210, within a range of .360 and 2.060. The original study had a Hedges’ *g* value of 1.510, within a range of .600–2.420. The effect size after removing samples does not differ significantly from the original study, and the total effect size interval after deletion is still within the total effect size interval of the original study, indicating that the meta‐analysis estimation results have high stability.

Moreover, a large mean difference is found in P3 amplitude (Hedges’ *g* = 1.382 *p* < .001, 95% CI = .654–2.109) with significantly higher amplitude in the IGD group than the control group. However, the mean difference is highly heterogeneous (*I* ^2^ = 86.535%, *Q* = 29.707, *p* < .001) indicating high between‐studies variation. Following this, we conducted a sensitivity analysis. In the analysis of the P3 EEG component, it was observed that the effect size from Littel et al. (2012) study deviated the most from other effect sizes, with Hedges’ *g* value of 2.899 and an effect size range of 2.114–3.683. Upon removing the data from this study, we found that the remaining four studies had an effect size with Hedges’ *g* value of 1.025, within a range of .529–1.522. The original study had a Hedges’ *g* value of 1.382, within a range of .654–2.109. The effect size after removing samples does not differ significantly from the original study, and the overall effect size range remained within the range of the original study. This indicates that the meta‐analysis estimates are stable and robust. Tables 2 and 3 illustrate graphical presentations of mean differences of N2 and P3, respectively.

**TABLE 2:** Forest plot of N2 amplitude.

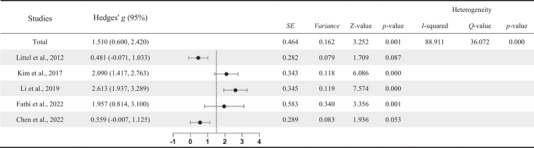

**TABLE 3:** Forest plot of P3 amplitude.

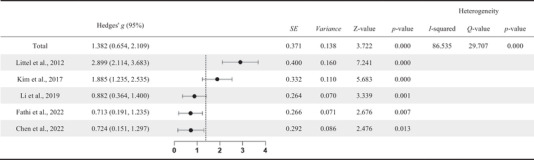

### 3.4 Publication bias

**FIGURE 4:**
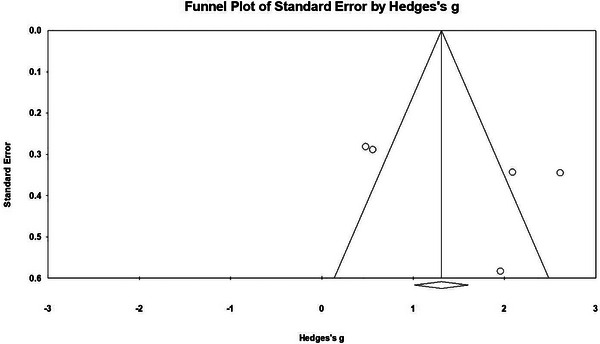
The funnel plots of N2.

For studies reporting on N2, the *Nfs* of N2 was 107, which is much greater than 5*k* + 10 = 35, so there is no publication bias. Similarly, Begg rank correlation test (*Z* = 1.225, *p* = .220) (*p* > .05) suggests the included studies were not affected by publication bias. The funnel plot shows that the effect size is concentrated below the graph and evenly distributed on both sides of the total effect, indicating that the publication bias is not obvious, and the total effect size estimation result is relatively reliable (refer to Figure 4).

**FIGURE 5:**
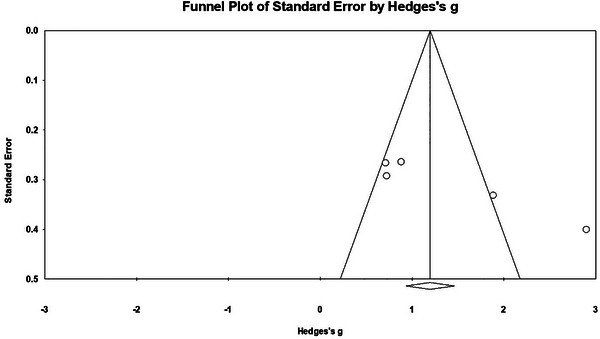
The funnel plots of P3.

For P3, no publication bias was detected as indicated by the fail‐safe N (*Nfs =* 115) and Begg and Mazumdar rank correlation (*Z* = 1.225, *p* = .221) (*p* > .05). The funnel graph shows that the effect size is concentrated on the bottom of the graph and evenly distributed on both sides of the total effect, suggesting that the effect of P3 is not affected by publication bias (refer to Figure 5). In summary, there is no publication bias for N2 and P3 in this study.

## 4 DISCUSSION

To our knowledge, this article is the first to examine the EEG components of inhibitory control functions in IGD using meta‐analysis. Findings highlight electrophysiological functioning of individuals with IGD differs significantly than those of non‐IGD. This is demonstrated by larger N2 and P3 amplitudes of individuals with IGD during inhibitory control tasks such as Go/NoGo and stop signal tasks.

A large N2 amplitude of ERP during inhibitory control tasks in pathological online gaming behavior has been associated with deficits in monitoring and suppression of nontarget stimuli (Folstein & Van Petten, 2008). Individuals with IGD may exhibit increased conflict monitoring due to the conflict between their desire to engage in gaming and the need to inhibit such behavior during the inhibitory control task. This is in line with the conflict monitoring hypothesis that the proposed N2 component represents the ability of the subjects to monitor the conflict process in the early stage of the task suppression and is the embodiment of the task monitoring ability (Folstein & Van Petten, 2008; Yeung et al., 2004). As a result, the heightened conflict monitoring can lead to larger N2 amplitudes in the IGD group (Dong et al., 2019; Kiefer et al., 1998). The large N2 component of ERP in the IGD group in the present review may therefore indicate that individuals with pathological online gaming behavior have excessive conflict monitoring processing of stimuli during the implementation of cognitive tasks (Liu et al., 2022; Rietdijk et al., 2014). Moreover, inhibition hypothesis holds that the N2 component reflects the ability of the subjects to suppress the processing process in the task (Folstein & Van Petten, 2008). The larger N2 component represents the lack of inhibition and control ability of the subjects, resulting in disinhibition behavior (Dustman et al., 1996). Individuals with IGD may experience deficits in cognitive control processes, such as inhibitory control. These deficits can manifest as increased difficulty in suppressing automatic responses and result in larger N2 amplitudes during inhibitory control tasks (Littel et al., 2019). Dysfunctional inhibitory control in IGD therefore may potentially be indicated by the inflated N2 amplitude in ERPs.

P3, as an assessment of stimuli during attention, is also commonly used as a comprehensive indicator of cognitive function (Polich, 2007; Rugg & Curran, 2007). In the inhibitory control task of IGD behavior, a large P3 amplitude of ERPs is associated with greater difficulty in controlling attention to cue‐related stimuli. Individuals with IGD may exhibit heightened attentional processing toward gaming‐related stimuli. This heightened attention can lead to increased neural activation and larger P3 amplitudes when processing relevant stimuli during inhibitory control tasks (Dong et al., 2013). Meanwhile, it is widely believed that P3 reflects the hyperarousal and increased emotional salience in the brain during information processing. Individuals with IGD often experience heightened physiological and emotional responses while engaging in gaming activities. This hyperarousal and increased emotional salience can lead to amplified neural responses and larger P3 amplitudes, reflecting the processing of emotionally salient stimuli during inhibitory control tasks (Dong et al., 2013). Moreover, P3 amplitudes indicate that the IGD had more difficulty than the controls in successfully inhibiting their response impulses. In the task, the subject needs to make the greatest effort, press the button quickly and accurately, and consume more cognitive resources. Compared with the control group, the internet addiction group needed extra cognitive processing resources when completing the task. Individuals with IGD may exhibit deficits in inhibitory control, leading to increased difficulty in suppressing automatic responses. For those with internet gaming disabilities, individuals with internet addiction show special and sensitive attention to internet content and have a strong psychological desire for the internet, and it is difficult to control their online behavior. The increased effort required to inhibit prepotent responses can result in larger P3 amplitudes during inhibitory control tasks (Brand et al., 2016). Therefore, inhibitory control of IGD may potentially be indicated by the inflated P3 amplitude in ERPs.

In addition, we found that in the meta‐analysis, the effect value of N2 was greater than that of P3 in the analysis of the EEG components of inhibition control in those with internet gaming disabilities. The reason for this phenomenon may be that, in the case of individual cognitive resources, subjects spent more cognitive resources on the monitoring and processing of the former component (N2 component) when completing the inhibition task. Less invested cognitive resources are consumed in the latter component (the P3 component) (Friedman & Miyake, 2004).

## 5 LIMITATIONS

This systematic review and meta‐analysis exhibit certain limitations, which should be taken into account. First, the cross‐sectional design of the included studies hampers our ability to draw causal inferences. Second, a relatively small sample size of only five studies was incorporated in this review, which may impact the effect size and contribute to heterogeneous results. Additionally, in real‐life scenarios, the likelihood of males exhibiting IGD symptoms is much higher than that of females, resulting in a scarcity of female participants in the IGD experiment. Most studies tend to have an unequal distribution of male and female participants in both the experimental and control groups. In the studies we included, the gender ratios in the experimental and control groups varied across different studies, which may be a major contributing factor to the observed heterogeneity in the research results. Lastly, this study exclusively focused on N2 and P3 brainwaves; however, future investigations may benefit from considering additional brainwaves associated with inhibitory control. This would encourage scholars to broaden their scope and conduct more research on the various brain electrical components related to inhibitory control.

## 6 FUTURE IMPLICATION AND CONCLUSION

First and foremost, longitudinal studies are needed to clarify the causal relationships between inhibitory control function and ERP components in individuals with IGD. Such research could help determine whether impaired inhibitory control is a cause or consequence of IGD, or whether the relationship is bidirectional. Understanding these relationships will be crucial for developing targeted prevention and intervention strategies for individuals at risk of or affected by IGD. It is also essential to consider potential moderators that may influence the relationship between IGD and inhibitory control, such as the severity of the disorder, comorbid psychiatric conditions, and individual differences in cognitive and neural functioning. By examining these moderators, researchers can gain a more comprehensive understanding of the factors that contribute to impaired inhibitory control in IGD and how these factors may interact with one another.

Second, in future research, we should strive to maintain a balanced gender ratio of IGD groups and HC groups included in the experiment. Moreover, in future experimental studies, researchers should focus more on conducting correlation analyses between EEG components and inhibitory control in individuals with IGD. This would contribute to further in‐depth research and extend the applicability of the meta‐analysis findings. Future research can also consider other EEG indicators related to inhibition control and adopt multiple inhibition control experimental paradigms, using various EEG devices to conduct EEG research on the inhibitory and control abilities of online game addicts, in order to expand this aspect of EEG research and provide more reference significance for EEG research in this direction.

Furthermore, to better understand the neurobiological underpinnings of IGD and inhibitory control, future research could also investigate the role of neurotransmitter systems, such as dopamine and serotonin, in the development and maintenance of the disorder. These neurotransmitter systems have been implicated in other addiction‐related disorders and may provide crucial insights into the mechanisms underlying IGD and its association with impaired inhibitory control.

Finally, future research should explore the role of individual differences in susceptibility to IGD, including genetic factors, personality traits, and environmental influences. Understanding these factors could help identify individuals who may be more vulnerable to developing gaming‐related problems and inform the development of targeted prevention and intervention programs. By examining the functioning of these systems in individuals with IGD, researchers could identify potential targets for pharmacological interventions aimed at improving cognitive functioning and reducing the severity of the disorder.

In conclusion, the findings of this study provide evidence for the utility of N2 and P3 amplitudes as indicators of inhibitory control in individuals with IGD. These findings support the neurophysiological basis for pathological gaming behavior as it has important implications for the assessment and diagnosis; N2 and P3 serve as neural markers in classifying the disorder. In light of these findings, it is essential to consider the potential implications for prevention and treatment strategies targeting IGD. Interventions targeting inhibitory function are expected to facilitate individuals with IGD to better regulate their gaming behavior and resist the urge to engage in excessive gaming.

## AUTHOR CONTRIBUTIONS

**Junjian Yu**: Conceptualization; methodology; software; data curation; investigation; writing—original draft; writing—review and editing; formal analysis; visualization. **Mohammad Farris Iman Leong Abdullah**: Writing—review and editing; supervision; project administration; resources. **Nor Shuhada Mansor**: Conceptualization; supervision; resources; project administration; writing—review and editing; validation.

## CONFLICT OF INTEREST STATEMENT

The authors declare that they have no conflicts of interest.

## FUNDING INFORMATION

The authors received no financial support for the research, authorship, and/or publication of this article.

### PEER REVIEW

The peer review history for this article is available at https://publons.com/publon/10.1002/brb3.3388.

## Data Availability

The data that support the findings of this study are available from the corresponding authors upon reasonable request.
